# Tetra­kis(4-amino­pyridine-κ*N* ^1^)dichlorido­copper(II) monohydrate

**DOI:** 10.1107/S1600536808015778

**Published:** 2008-06-07

**Authors:** Hoong-Kun Fun, A. Sinthya, Samuel Robinson Jebas, Suganthi Devadasan

**Affiliations:** a X-ray Crystallography Unit, School of Physics, Universiti Sains Malaysia, 11800 USM, Penang, Malaysia; b Department of Electronics, St Joseph’s College, Tiruchirappalli 620 001, India; c Department of Physics, Karunya University, Karunya Nagar, Coimbatore 641 114, India

## Abstract

The asymmetric unit of the title compound, [CuCl_2_(C_5_H_6_N_2_)_4_]·H_2_O, contains two crystallographically independent complex mol­ecules and two water mol­ecules. The Cu^II^ ion in each mol­ecule is six-coordinated in an elongated octa­hedral geometry, with the equatorial plane defined by four pyridine N atoms of four amino­pyridine ligands and the axial positions occupied by two Cl atoms. In the crystal structure, mol­ecules are linked into a three-dimensional framework by C—H⋯Cl, O—H⋯Cl, N—H⋯O, N—H⋯Cl and N—H⋯N hydrogen bonds and C/N—H⋯π inter­actions involving the pyridine rings.

## Related literature

For related literature on 4-amino­pyridine, see: Judge & Bever (2006 ▶); Schwid *et al.* (1997 ▶); Strupp *et al.* (2004 ▶). For bond lengths, see: Moncol *et al.* (2004 ▶); Zaleski *et al.* (2005 ▶); Anderson *et al.* (2005 ▶).
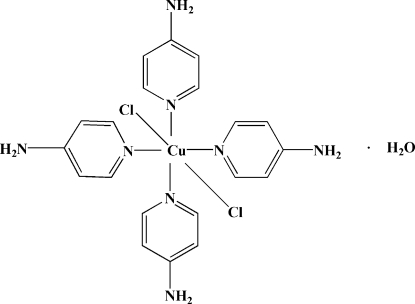


## Experimental

### 

#### Crystal data

[CuCl_2_(C_5_H_6_N_2_)_4_]·H_2_O
*M* *_r_* = 528.93
Triclinic, 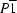

*a* = 9.5430 (2) Å
*b* = 14.1606 (2) Å
*c* = 17.4662 (3) Å
α = 88.463 (1)°
β = 86.075 (1)°
γ = 85.781 (1)°
*V* = 2347.81 (7) Å^3^
*Z* = 4
Mo *K*α radiation
μ = 1.19 mm^−1^
*T* = 100.0 (1) K
0.51 × 0.40 × 0.12 mm

#### Data collection

Bruker SMART APEXII CCD area-detector diffractometer
Absorption correction: multi-scan (*SADABS*; Bruker, 2005 ▶) *T*_min_ = 0.583, *T*_max_ = 0.871
106511 measured reflections
24484 independent reflections
17417 reflections with *I* > 2σ(*I*)
*R*_int_ = 0.047

#### Refinement

*R*[*F* ^2^ > 2σ(*F* ^2^)] = 0.041
*wR*(*F* ^2^) = 0.104
*S* = 1.07
24484 reflections
593 parameters
6 restraints
H atoms treated by a mixture of independent and constrained refinement
Δρ_max_ = 0.92 e Å^−3^
Δρ_min_ = −1.11 e Å^−3^

### 

Data collection: *APEX2* (Bruker, 2005 ▶); cell refinement: *APEX2*; data reduction: *SAINT* (Bruker, 2005 ▶); program(s) used to solve structure: *SHELXTL* (Sheldrick, 2008 ▶); program(s) used to refine structure: *SHELXTL*; molecular graphics: *SHELXTL*; software used to prepare material for publication: *SHELXTL* and *PLATON* (Spek, 2003 ▶).

## Supplementary Material

Crystal structure: contains datablocks global, I. DOI: 10.1107/S1600536808015778/ci2603sup1.cif

Structure factors: contains datablocks I. DOI: 10.1107/S1600536808015778/ci2603Isup2.hkl

Additional supplementary materials:  crystallographic information; 3D view; checkCIF report

## Figures and Tables

**Table 1:** Hydrogen-bond geometry (Å, °) *Cg*1, *Cg*2 and *Cg*3 are centroids of the N1/C1–C5, N9/C21–C25 and N11/C26–C30 rings, respectively.

*D*—H⋯*A*	*D*—H	H⋯*A*	*D*⋯*A*	*D*—H⋯*A*
C2—H2⋯Cl2^i^	0.93	2.68	3.5955 (13)	167
C6—H6⋯Cl2	0.93	2.80	3.3665 (13)	121
C10—H10⋯Cl1	0.93	2.78	3.4430 (13)	129
C20—H20⋯Cl1	0.93	2.64	3.3522 (13)	134
C25—H25⋯Cl4	0.93	2.71	3.2928 (13)	121
C26—H26⋯N9	0.93	2.62	3.0473 (17)	108
C35—H35⋯Cl4	0.93	2.67	3.3947 (14)	136
N4—H4*A*⋯O1*W*^ii^	0.86	2.38	3.2094 (17)	163
N4—H4*B*⋯Cl1^iii^	0.86	2.43	3.2893 (13)	175
N6—H6*A*⋯Cl4^iv^	0.86	2.82	3.4066 (13)	127
N8—H8*B*⋯Cl2^iv^	0.86	2.56	3.4021 (13)	166
N10—H10*B*⋯Cl3^v^	0.86	2.41	3.2596 (11)	171
N12—H12*A*⋯O1*W*	0.86	2.06	2.8908 (16)	162
N14—H14*B*⋯O1*W*^vi^	0.86	2.25	3.0103 (18)	147
N16—H16*B*⋯N16^vii^	0.86	2.52	3.2036 (17)	137
O1*W*—H1*W*1⋯Cl1^iii^	0.83 (1)	2.26 (1)	3.0614 (12)	163 (2)
O1*W*—H2*W*1⋯Cl4^viii^	0.83 (1)	2.26 (1)	3.0694 (12)	164 (2)
C37—H37⋯*Cg*1^ix^	0.93	2.81	3.5492 (14)	137
C39—H39⋯*Cg*1^x^	0.93	2.95	3.7306 (14)	142
N2—H2*B*⋯*Cg*2^xi^	0.86	2.75	3.3359 (13)	126
C12—H12⋯*Cg*3	0.93	2.79	3.6488 (14)	154
C14—H14⋯*Cg*3^iv^	0.93	2.83	3.6193 (13)	144

Symmetry codes: (i) 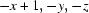; (ii) 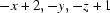; (iii) 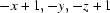; (iv) 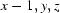; (v) 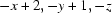; (vi) 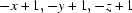; (vii) 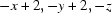; (viii) 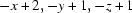; (ix) 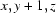; (x) 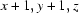; (xi) 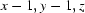.

**Fig. 1.**
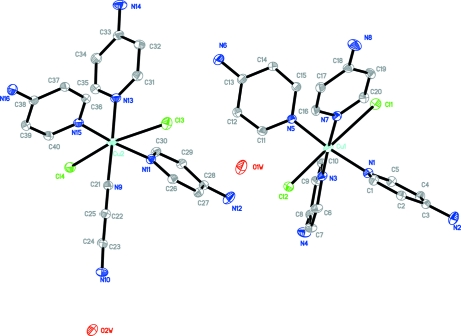
The asymmetric unit of the title compound, showing 50% probability displacement ellipsoids and the atom-numbering scheme. Hydrogen atoms are omitted for clarity.

**Fig. 2.**
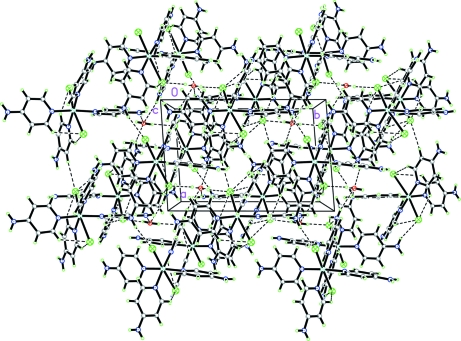
The crystal packing of the title compound, viewed along the c axis. Hydrogen bonds are shown as dashed lines.

## supplementary crystallographic information

### Comment

4-Aminopyridine (Fampridine) is used clinically in Lambert–Eaton myasthenic syndrome and multiple sclerosis because by blocking potassium channels it prolongs action potentials thereby increasing transmitter release at the neuromuscular junctions (Judge & Bever, 2006; Schwid *et al.*,1997; Strupp *et al.*, 2004).

The asymmetric unit of the title compound contains two crystallographically independent complex molecules and two water molecules (Fig. 1). The Cu^II^ ion in each molecule is six-coordinated in an elongated octahedral geometry formed by four pyridine N atoms from four aminopyridine ligands and two Cl atoms. The two Cl atoms are located at apical positions and the four N atoms form the basal plane. The Jahn–Teller elongation observed in Cu1—Cl1 [3.2185 (4) Å] and Cu2—Cl3 [3.1884 (4) Å] distances are consistent with those reported earlier (Moncol *et al.*, 2004). The average Cu—N bond length of 2.0205 (11) Å agree well with that reported for a copper complex (Zaleski *et al.*, 2005). The bond lengths and angles in the 4-aminopyridine units agree well with those reported earlier (Anderson *et al.*, 2005).

The crystal packing is consolidated by intermolecular O—H···Cl, N—H···O, N—H···N, N—H···Cl and C—H···Cl hydrogen bonds, and and C/N—H···π interactions (Table 2) involving the pyridine rings to form a three-dimensional framework.

### Experimental

A solution of 4-aminopyridine (0.376 g) in water (20 ml) was added to a solution of CuCl_2_.2H_2_O (0.170 g) in water (20 ml) and the mixture was stirred at 303 K for 6 h. The clear blue solution obtained was filtered and allowed to evaporate slowly. Blue crystals of the title compound were obtained after a month.

### Refinement

H atoms of the water molecules were located in a difference map and refined with O—H and H···H distance restraints of 0.84 (1) and 1.37 (2) Å, respectively. The remaining H atoms were positioned geometrically [C—H = 0.93 Å and N—H = 0.86 Å] and refined using a riding model, with *U*_iso_(H) = 1.2*U*_eq_(C,*N*)].

### Crystal data

**Table:**

[CuCl_2_(C_5_H_6_N_2_)_4_]·H_2_O	*Z* = 4
*M**_r_* = 528.93	*F*_000_ = 1092
Triclinic, *P*1	*D*_x_ = 1.496 Mg m^−^^3^
Hall symbol: -P 1	Mo *K*α radiation λ = 0.71073 Å
*a* = 9.5430 (2) Å	Cell parameters from 9885 reflections
*b* = 14.1606 (2) Å	θ = 2.5–34.6º
*c* = 17.4662 (3) Å	µ = 1.19 mm^−^^1^
α = 88.463 (1)º	*T* = 100.0 (1) K
β = 86.075 (1)º	Plate, purple
γ = 85.781 (1)º	0.51 × 0.40 × 0.12 mm
*V* = 2347.81 (7) Å^3^	

### Data collection

**Table:**

Bruker SMART APEXII CCD area-detector diffractometer	24484 independent reflections
Radiation source: fine-focus sealed tube	17417 reflections with *I* > 2σ(*I*)
Monochromator: graphite	*R*_int_ = 0.047
*T* = 100.0(1) K	θ_max_ = 37.5º
φ and ω scans	θ_min_ = 1.2º
Absorption correction: multi-scan(SADABS; Bruker, 2005)	*h* = −16→15
*T*_min_ = 0.583, *T*_max_ = 0.871	*k* = −23→24
106511 measured reflections	*l* = −29→29

### Refinement

**Table:**

Refinement on *F*^2^	Secondary atom site location: difference Fourier map
Least-squares matrix: full	Hydrogen site location: inferred from neighbouring sites
*R*[*F*^2^ > 2σ(*F*^2^)] = 0.041	H atoms treated by a mixture of independent and constrained refinement
*wR*(*F*^2^) = 0.104	*w* = 1/[σ^2^(*F*_o_^2^) + (0.0446*P*)^2^ + 0.3682*P*] where *P* = (*F*_o_^2^ + 2*F*_c_^2^)/3
*S* = 1.07	(Δ/σ)_max_ = 0.001
24484 reflections	Δρ_max_ = 0.92 e Å^−^^3^
593 parameters	Δρ_min_ = −1.11 e Å^−^^3^
6 restraints	Extinction correction: none
Primary atom site location: structure-invariant direct methods	

### Special details

**Table:**

Experimental. The data was collected with the Oxford Cyrosystem Cobra low-temperature attachment.
Geometry. All e.s.d.'s (except the e.s.d. in the dihedral angle between two l.s. planes) are estimated using the full covariance matrix. The cell e.s.d.'s are taken into account individually in the estimation of e.s.d.'s in distances, angles and torsion angles; correlations between e.s.d.'s in cell parameters are only used when they are defined by crystal symmetry. An approximate (isotropic) treatment of cell e.s.d.'s is used for estimating e.s.d.'s involving l.s. planes.
Refinement. Refinement of *F*^2^ against ALL reflections. The weighted *R*-factor *wR* and goodness of fit *S* are based on *F*^2^, conventional *R*-factors *R* are based on *F*, with *F* set to zero for negative *F*^2^. The threshold expression of *F*^2^ > σ(*F*^2^) is used only for calculating *R*-factors(gt) *etc*. and is not relevant to the choice of reflections for refinement. *R*-factors based on *F*^2^ are statistically about twice as large as those based on *F*, and *R*- factors based on ALL data will be even larger.

### Fractional atomic coordinates and isotropic or equivalent isotropic displacement parameters (Å^2^)

**Table:**

	*x*	*y*	*z*	*U*_iso_*/*U*_eq_	
Cu1	0.438175 (17)	0.078184 (11)	0.230903 (9)	0.01546 (4)	
N1	0.42808 (11)	−0.05177 (8)	0.18839 (6)	0.01482 (18)	
N2	0.37359 (14)	−0.31516 (8)	0.09807 (7)	0.0227 (2)	
H2A	0.3669	−0.3204	0.0495	0.027*	
H2B	0.3679	−0.3641	0.1281	0.027*	
N3	0.58224 (11)	0.02793 (7)	0.30393 (6)	0.01460 (18)	
N4	0.88631 (12)	−0.05810 (9)	0.45634 (7)	0.0204 (2)	
H4A	0.9675	−0.0825	0.4396	0.025*	
H4B	0.8678	−0.0508	0.5048	0.025*	
N5	0.42913 (11)	0.20525 (7)	0.28145 (6)	0.01477 (18)	
N6	0.40879 (14)	0.45962 (9)	0.39775 (8)	0.0280 (3)	
H6A	0.3293	0.4829	0.4178	0.034*	
H6B	0.4840	0.4882	0.4026	0.034*	
N7	0.27731 (11)	0.12641 (8)	0.16710 (6)	0.01593 (19)	
N8	−0.06992 (13)	0.23308 (10)	0.05134 (8)	0.0276 (3)	
H8A	−0.0617	0.2769	0.0167	0.033*	
H8B	−0.1504	0.2106	0.0623	0.033*	
C1	0.41925 (13)	−0.06352 (9)	0.11226 (7)	0.0155 (2)	
H1	0.4246	−0.0105	0.0799	0.019*	
C2	0.40285 (13)	−0.14884 (9)	0.07986 (7)	0.0154 (2)	
H2	0.3983	−0.1527	0.0270	0.018*	
C3	0.39299 (13)	−0.23054 (9)	0.12703 (7)	0.0160 (2)	
C4	0.40297 (14)	−0.21872 (9)	0.20635 (7)	0.0172 (2)	
H4	0.3979	−0.2704	0.2401	0.021*	
C5	0.42019 (13)	−0.13030 (9)	0.23353 (7)	0.0162 (2)	
H5	0.4268	−0.1243	0.2860	0.019*	
C6	0.70980 (14)	−0.01180 (9)	0.27928 (7)	0.0173 (2)	
H6	0.7287	−0.0193	0.2268	0.021*	
C7	0.81305 (14)	−0.04176 (10)	0.32694 (7)	0.0180 (2)	
H7	0.8989	−0.0688	0.3067	0.022*	
C8	0.78850 (13)	−0.03145 (9)	0.40679 (7)	0.0144 (2)	
C9	0.65482 (13)	0.00904 (9)	0.43265 (7)	0.0158 (2)	
H9	0.6324	0.0169	0.4849	0.019*	
C10	0.55757 (13)	0.03684 (9)	0.38032 (7)	0.0159 (2)	
H10	0.4700	0.0632	0.3987	0.019*	
C11	0.54513 (13)	0.25191 (9)	0.28928 (8)	0.0175 (2)	
H11	0.6310	0.2255	0.2685	0.021*	
C12	0.54381 (14)	0.33629 (9)	0.32624 (8)	0.0190 (2)	
H12	0.6270	0.3657	0.3300	0.023*	
C13	0.41553 (14)	0.37788 (9)	0.35848 (8)	0.0176 (2)	
C14	0.29433 (13)	0.32995 (9)	0.34973 (8)	0.0168 (2)	
H14	0.2069	0.3549	0.3696	0.020*	
C15	0.30574 (13)	0.24571 (9)	0.31143 (7)	0.0159 (2)	
H15	0.2242	0.2151	0.3059	0.019*	
C16	0.28786 (14)	0.19626 (9)	0.11362 (8)	0.0180 (2)	
H16	0.3750	0.2209	0.1035	0.022*	
C17	0.17711 (14)	0.23325 (9)	0.07308 (8)	0.0190 (2)	
H17	0.1910	0.2803	0.0357	0.023*	
C18	0.04303 (13)	0.19955 (10)	0.08841 (8)	0.0179 (2)	
C19	0.03170 (14)	0.12759 (10)	0.14505 (7)	0.0180 (2)	
H19	−0.0549	0.1034	0.1581	0.022*	
C20	0.14907 (14)	0.09318 (9)	0.18106 (7)	0.0175 (2)	
H20	0.1395	0.0442	0.2171	0.021*	
Cu2	0.926147 (17)	0.588239 (11)	0.265503 (9)	0.01796 (4)	
N9	1.07098 (11)	0.53919 (8)	0.18340 (6)	0.01519 (19)	
N10	1.36478 (12)	0.42921 (8)	0.01531 (6)	0.0183 (2)	
H10A	1.4401	0.3975	0.0283	0.022*	
H10B	1.3490	0.4372	−0.0324	0.022*	
N11	0.91724 (11)	0.45781 (8)	0.31403 (6)	0.01547 (19)	
N12	0.91541 (14)	0.18712 (8)	0.41402 (7)	0.0248 (3)	
H12A	0.9061	0.1801	0.4631	0.030*	
H12B	0.9251	0.1382	0.3855	0.030*	
N13	0.77568 (12)	0.64322 (8)	0.34276 (6)	0.0170 (2)	
N14	0.45970 (14)	0.77011 (10)	0.49076 (7)	0.0262 (3)	
H14A	0.4812	0.8030	0.5284	0.031*	
H14B	0.3728	0.7633	0.4835	0.031*	
N15	0.91270 (11)	0.71282 (8)	0.20637 (6)	0.01664 (19)	
N16	0.89456 (13)	0.95381 (8)	0.06323 (7)	0.0204 (2)	
H16A	0.8173	0.9718	0.0428	0.024*	
H16B	0.9680	0.9850	0.0537	0.024*	
C21	1.05091 (13)	0.55075 (9)	0.10788 (7)	0.0157 (2)	
H21	0.9680	0.5832	0.0939	0.019*	
C22	1.14589 (13)	0.51732 (9)	0.05055 (7)	0.0156 (2)	
H22	1.1273	0.5283	−0.0006	0.019*	
C23	1.27166 (12)	0.46630 (9)	0.06954 (7)	0.0141 (2)	
C24	1.29340 (13)	0.45489 (9)	0.14834 (7)	0.0164 (2)	
H24	1.3753	0.4228	0.1642	0.020*	
C25	1.19284 (14)	0.49147 (9)	0.20156 (7)	0.0167 (2)	
H25	1.2094	0.4830	0.2532	0.020*	
C26	0.92843 (13)	0.37962 (9)	0.27115 (7)	0.0165 (2)	
H26	0.9361	0.3873	0.2180	0.020*	
C27	0.92913 (14)	0.28925 (9)	0.30160 (7)	0.0171 (2)	
H27	0.9389	0.2377	0.2693	0.021*	
C28	0.91497 (13)	0.27475 (9)	0.38181 (7)	0.0164 (2)	
C29	0.89945 (13)	0.35662 (9)	0.42649 (7)	0.0164 (2)	
H29	0.8873	0.3515	0.4797	0.020*	
C30	0.90246 (13)	0.44415 (9)	0.39074 (7)	0.0164 (2)	
H30	0.8938	0.4973	0.4214	0.020*	
C31	0.63836 (14)	0.63335 (10)	0.33431 (8)	0.0185 (2)	
H31	0.6150	0.5965	0.2944	0.022*	
C32	0.53041 (14)	0.67467 (10)	0.38134 (7)	0.0182 (2)	
H32	0.4372	0.6666	0.3723	0.022*	
C33	0.56243 (14)	0.72915 (9)	0.44304 (7)	0.0177 (2)	
C34	0.70551 (15)	0.73799 (10)	0.45233 (8)	0.0210 (2)	
H34	0.7323	0.7723	0.4929	0.025*	
C35	0.80628 (14)	0.69599 (10)	0.40169 (8)	0.0196 (2)	
H35	0.9004	0.7044	0.4084	0.024*	
C36	0.79327 (13)	0.74421 (9)	0.17431 (8)	0.0180 (2)	
H36	0.7138	0.7105	0.1845	0.022*	
C37	0.78247 (13)	0.82367 (9)	0.12713 (8)	0.0174 (2)	
H37	0.6975	0.8426	0.1063	0.021*	
C38	0.90066 (13)	0.87553 (9)	0.11090 (7)	0.0160 (2)	
C39	1.02509 (14)	0.84304 (9)	0.14474 (8)	0.0181 (2)	
H39	1.1061	0.8755	0.1359	0.022*	
C40	1.02618 (13)	0.76286 (9)	0.19099 (8)	0.0179 (2)	
H40	1.1096	0.7422	0.2127	0.021*	
Cl1	0.20362 (3)	0.01980 (2)	0.361362 (18)	0.01910 (6)	
Cl2	0.64240 (3)	0.12291 (2)	0.122477 (17)	0.01617 (5)	
Cl3	0.69715 (4)	0.51337 (2)	0.161801 (18)	0.02111 (6)	
Cl4	1.14798 (3)	0.62545 (2)	0.353735 (19)	0.01962 (6)	
O1W	0.82107 (13)	0.18216 (8)	0.57474 (6)	0.0285 (2)	
O2W	1.62468 (11)	0.33858 (7)	0.07154 (6)	0.02102 (19)	
H1W1	0.810 (2)	0.1333 (8)	0.6007 (10)	0.037 (6)*	
H2W1	0.834 (2)	0.2276 (9)	0.6020 (10)	0.039 (6)*	
H1W2	1.628 (3)	0.2884 (9)	0.0979 (11)	0.052 (7)*	
H2W2	1.648 (2)	0.3826 (10)	0.0992 (11)	0.048 (7)*	

### Atomic displacement parameters (Å^2^)

**Table:**

	*U*^11^	*U*^22^	*U*^33^	*U*^12^	*U*^13^	*U*^23^
Cu1	0.01684 (7)	0.01299 (7)	0.01708 (7)	0.00168 (5)	−0.00697 (5)	−0.00320 (5)
N1	0.0164 (5)	0.0146 (5)	0.0136 (4)	0.0001 (4)	−0.0033 (3)	−0.0015 (3)
N2	0.0330 (6)	0.0165 (5)	0.0193 (5)	−0.0048 (5)	−0.0016 (5)	−0.0046 (4)
N3	0.0160 (4)	0.0138 (4)	0.0141 (4)	0.0008 (4)	−0.0033 (3)	−0.0021 (3)
N4	0.0158 (5)	0.0302 (6)	0.0149 (5)	0.0041 (4)	−0.0036 (4)	−0.0019 (4)
N5	0.0137 (4)	0.0139 (5)	0.0170 (5)	0.0003 (4)	−0.0035 (3)	−0.0029 (3)
N6	0.0220 (6)	0.0183 (6)	0.0442 (8)	−0.0008 (5)	−0.0034 (5)	−0.0124 (5)
N7	0.0162 (5)	0.0144 (5)	0.0177 (5)	−0.0003 (4)	−0.0053 (4)	−0.0017 (3)
N8	0.0167 (5)	0.0331 (7)	0.0325 (7)	0.0028 (5)	−0.0063 (5)	0.0052 (5)
C1	0.0161 (5)	0.0158 (5)	0.0147 (5)	−0.0011 (4)	−0.0026 (4)	−0.0002 (4)
C2	0.0153 (5)	0.0182 (5)	0.0128 (5)	−0.0008 (4)	−0.0019 (4)	−0.0026 (4)
C3	0.0152 (5)	0.0155 (5)	0.0174 (5)	−0.0013 (4)	−0.0004 (4)	−0.0027 (4)
C4	0.0210 (6)	0.0149 (5)	0.0156 (5)	−0.0019 (4)	−0.0006 (4)	0.0003 (4)
C5	0.0178 (5)	0.0168 (5)	0.0140 (5)	−0.0011 (4)	−0.0024 (4)	−0.0006 (4)
C6	0.0202 (6)	0.0185 (6)	0.0126 (5)	0.0040 (4)	−0.0020 (4)	−0.0032 (4)
C7	0.0173 (5)	0.0215 (6)	0.0146 (5)	0.0044 (5)	−0.0013 (4)	−0.0035 (4)
C8	0.0145 (5)	0.0150 (5)	0.0139 (5)	−0.0009 (4)	−0.0027 (4)	−0.0010 (4)
C9	0.0151 (5)	0.0194 (6)	0.0129 (5)	−0.0009 (4)	−0.0004 (4)	−0.0016 (4)
C10	0.0142 (5)	0.0170 (5)	0.0164 (5)	0.0001 (4)	−0.0014 (4)	−0.0017 (4)
C11	0.0137 (5)	0.0180 (6)	0.0208 (6)	0.0003 (4)	−0.0023 (4)	−0.0026 (4)
C12	0.0149 (5)	0.0165 (6)	0.0262 (6)	−0.0019 (4)	−0.0033 (4)	−0.0036 (4)
C13	0.0188 (5)	0.0138 (5)	0.0202 (6)	0.0003 (4)	−0.0030 (4)	−0.0021 (4)
C14	0.0146 (5)	0.0153 (5)	0.0203 (6)	0.0006 (4)	−0.0007 (4)	−0.0012 (4)
C15	0.0140 (5)	0.0150 (5)	0.0191 (6)	−0.0007 (4)	−0.0032 (4)	−0.0013 (4)
C16	0.0153 (5)	0.0155 (5)	0.0238 (6)	−0.0032 (4)	−0.0044 (4)	0.0006 (4)
C17	0.0177 (5)	0.0158 (6)	0.0239 (6)	−0.0009 (4)	−0.0051 (5)	0.0024 (4)
C18	0.0144 (5)	0.0191 (6)	0.0201 (6)	0.0029 (4)	−0.0045 (4)	−0.0034 (4)
C19	0.0156 (5)	0.0224 (6)	0.0163 (6)	−0.0037 (4)	−0.0010 (4)	−0.0027 (4)
C20	0.0193 (6)	0.0189 (6)	0.0149 (5)	−0.0032 (4)	−0.0023 (4)	−0.0010 (4)
Cu2	0.01974 (8)	0.01387 (7)	0.01829 (8)	0.00360 (6)	0.00663 (6)	0.00303 (5)
N9	0.0154 (4)	0.0139 (4)	0.0155 (5)	0.0015 (4)	0.0009 (3)	0.0007 (3)
N10	0.0168 (5)	0.0225 (5)	0.0146 (5)	0.0031 (4)	0.0012 (4)	−0.0009 (4)
N11	0.0175 (5)	0.0137 (4)	0.0149 (5)	−0.0004 (4)	0.0000 (4)	0.0007 (3)
N12	0.0378 (7)	0.0138 (5)	0.0215 (6)	0.0015 (5)	0.0031 (5)	0.0021 (4)
N13	0.0177 (5)	0.0149 (5)	0.0174 (5)	0.0012 (4)	0.0034 (4)	0.0004 (4)
N14	0.0234 (6)	0.0300 (7)	0.0239 (6)	0.0050 (5)	0.0040 (5)	−0.0088 (5)
N15	0.0157 (5)	0.0145 (5)	0.0186 (5)	0.0015 (4)	0.0030 (4)	0.0015 (4)
N16	0.0218 (5)	0.0163 (5)	0.0226 (6)	−0.0012 (4)	0.0002 (4)	0.0039 (4)
C21	0.0144 (5)	0.0153 (5)	0.0172 (5)	0.0003 (4)	−0.0006 (4)	0.0002 (4)
C22	0.0155 (5)	0.0172 (5)	0.0144 (5)	−0.0011 (4)	−0.0020 (4)	−0.0008 (4)
C23	0.0137 (5)	0.0129 (5)	0.0156 (5)	−0.0017 (4)	0.0002 (4)	−0.0013 (4)
C24	0.0161 (5)	0.0157 (5)	0.0169 (5)	0.0027 (4)	−0.0018 (4)	−0.0002 (4)
C25	0.0192 (5)	0.0156 (5)	0.0147 (5)	0.0021 (4)	0.0000 (4)	0.0006 (4)
C26	0.0176 (5)	0.0172 (5)	0.0144 (5)	−0.0005 (4)	0.0001 (4)	−0.0015 (4)
C27	0.0185 (5)	0.0143 (5)	0.0184 (6)	−0.0003 (4)	0.0003 (4)	−0.0030 (4)
C28	0.0153 (5)	0.0150 (5)	0.0186 (6)	−0.0003 (4)	−0.0005 (4)	0.0009 (4)
C29	0.0185 (5)	0.0159 (5)	0.0143 (5)	0.0004 (4)	0.0000 (4)	0.0005 (4)
C30	0.0189 (5)	0.0148 (5)	0.0153 (5)	0.0002 (4)	−0.0003 (4)	−0.0013 (4)
C31	0.0201 (6)	0.0193 (6)	0.0156 (6)	−0.0007 (5)	0.0018 (4)	−0.0017 (4)
C32	0.0173 (5)	0.0207 (6)	0.0159 (6)	0.0006 (5)	0.0007 (4)	−0.0008 (4)
C33	0.0207 (6)	0.0152 (5)	0.0161 (5)	0.0018 (4)	0.0029 (4)	−0.0001 (4)
C34	0.0226 (6)	0.0208 (6)	0.0199 (6)	−0.0021 (5)	−0.0011 (5)	−0.0051 (5)
C35	0.0180 (6)	0.0182 (6)	0.0226 (6)	−0.0016 (5)	0.0003 (5)	−0.0008 (4)
C36	0.0145 (5)	0.0154 (5)	0.0237 (6)	−0.0011 (4)	0.0011 (4)	0.0000 (4)
C37	0.0146 (5)	0.0154 (5)	0.0219 (6)	0.0000 (4)	−0.0011 (4)	0.0011 (4)
C38	0.0179 (5)	0.0138 (5)	0.0156 (5)	0.0003 (4)	0.0017 (4)	−0.0004 (4)
C39	0.0160 (5)	0.0172 (6)	0.0210 (6)	−0.0026 (4)	0.0010 (4)	0.0003 (4)
C40	0.0143 (5)	0.0174 (6)	0.0215 (6)	0.0008 (4)	0.0000 (4)	0.0006 (4)
Cl1	0.02220 (14)	0.01924 (14)	0.01585 (13)	−0.00149 (11)	−0.00145 (10)	0.00041 (10)
Cl2	0.01525 (12)	0.01728 (13)	0.01575 (13)	−0.00059 (10)	−0.00079 (9)	0.00145 (9)
Cl3	0.02782 (16)	0.02046 (14)	0.01555 (13)	−0.00381 (12)	−0.00212 (11)	−0.00224 (10)
Cl4	0.01772 (13)	0.02048 (14)	0.02104 (14)	−0.00009 (11)	−0.00386 (10)	−0.00442 (11)
O1W	0.0419 (6)	0.0194 (5)	0.0242 (5)	−0.0066 (5)	0.0045 (5)	−0.0017 (4)
O2W	0.0255 (5)	0.0154 (4)	0.0226 (5)	0.0002 (4)	−0.0064 (4)	−0.0015 (4)

### Geometric parameters (Å, °)

**Table:**

Cu1—N1	2.0140 (11)	Cu2—N9	2.0224 (10)
Cu1—N3	2.0177 (10)	Cu2—N13	2.0286 (11)
Cu1—N5	2.0190 (11)	Cu2—Cl4	2.7907 (4)
Cu1—N7	2.0258 (11)	Cu2—Cl3	3.1884 (4)
Cu1—Cl2	2.7199 (3)	N9—C21	1.3498 (16)
Cu1—Cl1	3.2185 (4)	N9—C25	1.3539 (16)
N1—C5	1.3494 (16)	N10—C23	1.3435 (16)
N1—C1	1.3536 (16)	N10—H10A	0.86
N2—C3	1.3431 (17)	N10—H10B	0.86
N2—H2A	0.86	N11—C26	1.3469 (17)
N2—H2B	0.86	N11—C30	1.3478 (16)
N3—C10	1.3466 (16)	N12—C28	1.3485 (17)
N3—C6	1.3506 (16)	N12—H12A	0.86
N4—C8	1.3431 (16)	N12—H12B	0.86
N4—H4A	0.86	N13—C31	1.3465 (18)
N4—H4B	0.86	N13—C35	1.3482 (18)
N5—C11	1.3468 (17)	N14—C33	1.3502 (17)
N5—C15	1.3496 (16)	N14—H14A	0.86
N6—C13	1.3564 (18)	N14—H14B	0.86
N6—H6A	0.86	N15—C36	1.3451 (17)
N6—H6B	0.86	N15—C40	1.3453 (17)
N7—C16	1.3466 (17)	N16—C38	1.3685 (17)
N7—C20	1.3477 (17)	N16—H16A	0.86
N8—C18	1.3455 (17)	N16—H16B	0.86
N8—H8A	0.86	C21—C22	1.3735 (17)
N8—H8B	0.86	C21—H21	0.93
C1—C2	1.3716 (18)	C22—C23	1.4095 (17)
C1—H1	0.93	C22—H22	0.93
C2—C3	1.4073 (18)	C23—C24	1.4092 (18)
C2—H2	0.93	C24—C25	1.3726 (17)
C3—C4	1.4104 (18)	C24—H24	0.93
C4—C5	1.3757 (18)	C25—H25	0.93
C4—H4	0.93	C26—C27	1.3723 (18)
C5—H5	0.93	C26—H26	0.93
C6—C7	1.3695 (18)	C27—C28	1.4091 (18)
C6—H6	0.93	C27—H27	0.93
C7—C8	1.4080 (17)	C28—C29	1.4080 (18)
C7—H7	0.93	C29—C30	1.3742 (18)
C8—C9	1.4098 (17)	C29—H29	0.93
C9—C10	1.3765 (18)	C30—H30	0.93
C9—H9	0.93	C31—C32	1.3783 (18)
C10—H10	0.93	C31—H31	0.93
C11—C12	1.3721 (19)	C32—C33	1.4050 (19)
C11—H11	0.93	C32—H32	0.93
C12—C13	1.4071 (18)	C33—C34	1.401 (2)
C12—H12	0.93	C34—C35	1.3742 (19)
C13—C14	1.4025 (18)	C34—H34	0.93
C14—C15	1.3770 (18)	C35—H35	0.93
C14—H14	0.93	C36—C37	1.3786 (18)
C15—H15	0.93	C36—H36	0.93
C16—C17	1.3762 (18)	C37—C38	1.3996 (18)
C16—H16	0.93	C37—H37	0.93
C17—C18	1.4044 (19)	C38—C39	1.4043 (19)
C17—H17	0.93	C39—C40	1.3755 (18)
C18—C19	1.4057 (19)	C39—H39	0.93
C19—C20	1.3738 (19)	C40—H40	0.93
C19—H19	0.93	O1W—H1W1	0.827 (9)
C20—H20	0.93	O1W—H2W1	0.833 (9)
Cu2—N11	2.0165 (11)	O2W—H1W2	0.835 (9)
Cu2—N15	2.0206 (11)	O2W—H2W2	0.849 (9)
			
N1—Cu1—N3	91.10 (4)	N11—Cu2—N9	91.26 (4)
N1—Cu1—N5	173.52 (4)	N15—Cu2—N9	88.17 (4)
N3—Cu1—N5	89.52 (4)	N11—Cu2—N13	91.71 (4)
N1—Cu1—N7	89.32 (4)	N15—Cu2—N13	88.57 (4)
N3—Cu1—N7	173.70 (4)	N9—Cu2—N13	176.35 (4)
N5—Cu1—N7	89.35 (4)	N11—Cu2—Cl4	90.90 (3)
N1—Cu1—Cl2	92.32 (3)	N15—Cu2—Cl4	97.84 (3)
N3—Cu1—Cl2	91.83 (3)	N9—Cu2—Cl4	88.13 (3)
N5—Cu1—Cl2	94.11 (3)	N13—Cu2—Cl4	93.94 (3)
N7—Cu1—Cl2	94.43 (3)	N11—Cu2—Cl3	82.30 (3)
N1—Cu1—Cl1	86.84 (3)	N15—Cu2—Cl3	88.92 (3)
N3—Cu1—Cl1	86.48 (3)	N9—Cu2—Cl3	86.13 (3)
N5—Cu1—Cl1	86.76 (3)	N13—Cu2—Cl3	92.17 (3)
N7—Cu1—Cl1	87.27 (3)	Cl4—Cu2—Cl3	170.991 (11)
Cl2—Cu1—Cl1	178.098 (10)	C21—N9—C25	116.39 (11)
C5—N1—C1	116.35 (11)	C21—N9—Cu2	122.13 (8)
C5—N1—Cu1	122.70 (9)	C25—N9—Cu2	121.47 (9)
C1—N1—Cu1	120.80 (9)	C23—N10—H10A	120.0
C3—N2—H2A	120.0	C23—N10—H10B	120.0
C3—N2—H2B	120.0	H10A—N10—H10B	120.0
H2A—N2—H2B	120.0	C26—N11—C30	116.62 (11)
C10—N3—C6	116.61 (11)	C26—N11—Cu2	121.42 (9)
C10—N3—Cu1	120.95 (8)	C30—N11—Cu2	121.96 (9)
C6—N3—Cu1	122.37 (9)	C28—N12—H12A	120.0
C8—N4—H4A	120.0	C28—N12—H12B	120.0
C8—N4—H4B	120.0	H12A—N12—H12B	120.0
H4A—N4—H4B	120.0	C31—N13—C35	116.72 (11)
C11—N5—C15	116.96 (11)	C31—N13—Cu2	120.80 (9)
C11—N5—Cu1	122.09 (8)	C35—N13—Cu2	122.35 (9)
C15—N5—Cu1	120.91 (9)	C33—N14—H14A	120.0
C13—N6—H6A	120.0	C33—N14—H14B	120.0
C13—N6—H6B	120.0	H14A—N14—H14B	120.0
H6A—N6—H6B	120.0	C36—N15—C40	117.21 (11)
C16—N7—C20	116.54 (11)	C36—N15—Cu2	121.08 (9)
C16—N7—Cu1	123.33 (9)	C40—N15—Cu2	121.39 (9)
C20—N7—Cu1	119.96 (9)	C38—N16—H16A	120.0
C18—N8—H8A	120.0	C38—N16—H16B	120.0
C18—N8—H8B	120.0	H16A—N16—H16B	120.0
H8A—N8—H8B	120.0	N9—C21—C22	123.79 (11)
N1—C1—C2	124.04 (12)	N9—C21—H21	118.1
N1—C1—H1	118.0	C22—C21—H21	118.1
C2—C1—H1	118.0	C21—C22—C23	119.77 (11)
C1—C2—C3	119.61 (11)	C21—C22—H22	120.1
C1—C2—H2	120.2	C23—C22—H22	120.1
C3—C2—H2	120.2	N10—C23—C24	121.81 (11)
N2—C3—C2	121.70 (12)	N10—C23—C22	121.67 (11)
N2—C3—C4	121.76 (12)	C24—C23—C22	116.51 (11)
C2—C3—C4	116.53 (11)	C25—C24—C23	119.55 (11)
C5—C4—C3	119.69 (12)	C25—C24—H24	120.2
C5—C4—H4	120.2	C23—C24—H24	120.2
C3—C4—H4	120.2	N9—C25—C24	123.97 (12)
N1—C5—C4	123.77 (12)	N9—C25—H25	118.0
N1—C5—H5	118.1	C24—C25—H25	118.0
C4—C5—H5	118.1	N11—C26—C27	123.57 (12)
N3—C6—C7	123.97 (12)	N11—C26—H26	118.2
N3—C6—H6	118.0	C27—C26—H26	118.2
C7—C6—H6	118.0	C26—C27—C28	119.91 (12)
C6—C7—C8	119.66 (12)	C26—C27—H27	120.0
C6—C7—H7	120.2	C28—C27—H27	120.0
C8—C7—H7	120.2	N12—C28—C29	121.79 (12)
N4—C8—C7	122.44 (11)	N12—C28—C27	121.76 (12)
N4—C8—C9	121.15 (11)	C29—C28—C27	116.45 (11)
C7—C8—C9	116.41 (11)	C30—C29—C28	119.36 (12)
C10—C9—C8	119.74 (11)	C30—C29—H29	120.3
C10—C9—H9	120.1	C28—C29—H29	120.3
C8—C9—H9	120.1	N11—C30—C29	124.06 (12)
N3—C10—C9	123.60 (11)	N11—C30—H30	118.0
N3—C10—H10	118.2	C29—C30—H30	118.0
C9—C10—H10	118.2	N13—C31—C32	123.80 (13)
N5—C11—C12	123.69 (12)	N13—C31—H31	118.1
N5—C11—H11	118.2	C32—C31—H31	118.1
C12—C11—H11	118.2	C31—C32—C33	119.43 (13)
C11—C12—C13	119.42 (12)	C31—C32—H32	120.3
C11—C12—H12	120.3	C33—C32—H32	120.3
C13—C12—H12	120.3	N14—C33—C34	122.24 (13)
N6—C13—C14	121.09 (12)	N14—C33—C32	121.20 (13)
N6—C13—C12	121.88 (12)	C34—C33—C32	116.56 (12)
C14—C13—C12	117.02 (12)	C35—C34—C33	120.14 (13)
C15—C14—C13	119.50 (11)	C35—C34—H34	119.9
C15—C14—H14	120.2	C33—C34—H34	119.9
C13—C14—H14	120.2	N13—C35—C34	123.33 (13)
N5—C15—C14	123.41 (12)	N13—C35—H35	118.3
N5—C15—H15	118.3	C34—C35—H35	118.3
C14—C15—H15	118.3	N15—C36—C37	123.47 (12)
N7—C16—C17	123.80 (12)	N15—C36—H36	118.3
N7—C16—H16	118.1	C37—C36—H36	118.3
C17—C16—H16	118.1	C36—C37—C38	119.33 (12)
C16—C17—C18	119.65 (12)	C36—C37—H37	120.3
C16—C17—H17	120.2	C38—C37—H37	120.3
C18—C17—H17	120.2	N16—C38—C37	120.96 (12)
N8—C18—C17	122.71 (13)	N16—C38—C39	121.87 (12)
N8—C18—C19	120.80 (13)	C37—C38—C39	117.17 (11)
C17—C18—C19	116.49 (12)	C40—C39—C38	119.49 (12)
C20—C19—C18	119.76 (12)	C40—C39—H39	120.3
C20—C19—H19	120.1	C38—C39—H39	120.3
C18—C19—H19	120.1	N15—C40—C39	123.33 (12)
N7—C20—C19	123.73 (12)	N15—C40—H40	118.3
N7—C20—H20	118.1	C39—C40—H40	118.3
C19—C20—H20	118.1	H1W1—O1W—H2W1	111.7 (14)
N11—Cu2—N15	171.22 (5)	H1W2—O2W—H2W2	107.8 (13)

### Hydrogen-bond geometry (Å, °)

**Table:**

*D*—H···*A*	*D*—H	H···*A*	*D*···*A*	*D*—H···*A*
C2—H2···Cl2^i^	0.93	2.68	3.5955 (13)	167
C6—H6···Cl2	0.93	2.80	3.3665 (13)	121
C10—H10···Cl1	0.93	2.78	3.4430 (13)	129
C20—H20···Cl1	0.93	2.64	3.3522 (13)	134
C25—H25···Cl4	0.93	2.71	3.2928 (13)	121
C26—H26···N9	0.93	2.62	3.0473 (17)	108
C35—H35···Cl4	0.93	2.67	3.3947 (14)	136
N4—H4A···O1W^ii^	0.86	2.38	3.2094 (17)	163
N4—H4B···Cl1^iii^	0.86	2.43	3.2893 (13)	175
N6—H6A···Cl4^iv^	0.86	2.82	3.4066 (13)	127
N8—H8B···Cl2^iv^	0.86	2.56	3.4021 (13)	166
N10—H10B···Cl3^v^	0.86	2.41	3.2596 (11)	171
N12—H12A···O1W	0.86	2.06	2.8908 (16)	162
N14—H14B···O1W^vi^	0.86	2.25	3.0103 (18)	147
N16—H16B···N16^vii^	0.86	2.52	3.2036 (17)	137
O1W—H1W1···Cl1^iii^	0.83 (1)	2.26 (1)	3.0614 (12)	163 (2)
O1W—H2W1···Cl4^viii^	0.83 (1)	2.26 (1)	3.0694 (12)	164 (2)
C37—H37···Cg1^ix^	0.93	2.81	3.5492 (14)	137
C39—H39···Cg1^x^	0.93	2.95	3.7306 (14)	142
N2—H2B···Cg2^xi^	0.86	2.75	3.3359 (13)	126
C12—H12···Cg3	0.93	2.79	3.6488 (14)	154
C14—H14···Cg3^iv^	0.93	2.83	3.6193 (13)	144

Symmetry codes: (i) −*x*+1, −*y*, −*z*; (ii) −*x*+2, −*y*, −*z*+1; (iii) −*x*+1, −*y*, −*z*+1; (iv) *x*−1, *y*, *z*; (v) −*x*+2, −*y*+1, −*z*; (vi) −*x*+1, −*y*+1, −*z*+1; (vii) −*x*+2, −*y*+2, −*z*; (viii) −*x*+2, −*y*+1, −*z*+1; (ix) *x*, *y*+1, *z*; (x) *x*+1, *y*+1, *z*; (xi) *x*−1, *y*−1, *z*.
